# Serotype influences on dengue severity: a cross-sectional study on 485 confirmed dengue cases in Vitória, Brazil

**DOI:** 10.1186/s12879-016-1668-y

**Published:** 2016-07-08

**Authors:** Creuza Rachel Vicente, Karl-Heinz Herbinger, Günter Fröschl, Camila Malta Romano, Aline de Souza Areias Cabidelle, Crispim Cerutti Junior

**Affiliations:** Center for International Health, Medical Center of the University of Munich, Leopoldstraße 7, 80802 Munich, Germany; Department of Infectious Diseases and Tropical Medicine, Medical Center of the University of Munich, Leopoldstraße 5, 80802 Munich, Germany; Institute of Tropical Medicine, University of São Paulo, Avenida Eneas de Carvalho Aguiar 470, 05403-000 São Paulo, Brazil; Coordination of Epidemiological Surveillance, Health Department of Vitória, Avenida Marechal Mascarenhas de Moraes 1185, 29017-010 Vitória, Brazil; Department of Social Medicine, Federal University of Espírito Santo, Avenida Marechal Campos 1468, 29040-090 Vitória, Brazil

**Keywords:** Dengue virus, Severe dengue, Signs and symptoms

## Abstract

**Background:**

Dengue is caused by a RNA virus of the family *Flaviviridae*, which presents four serotypes (DENV-1 to DENV-4) capable of inducing hemorrhage. The purpose of this study was to evaluate the influence of serotype on the outcome of dengue.

**Methods:**

This cross-sectional study included data from dengue cases with serotyping results that occurred between 2009 and 2013 in Vitória, Espírito Santo, Brazil. Data were accessed through the Information System for Notifiable Diseases. Chi-square test, Fisher exact test, Mann–Whitney *U* test, and logistic regression were performed to assess associations between different serotypes and dengue severity, while considering gender and age.

**Results:**

The sample consisted of 485 laboratory confirmed dengue cases, of which 46.4 % were females, with median age of 26 years. Regarding overall samples, 77.3 % were caused by DENV-1, 16.1 % by DENV-4, 6.4 % by DENV-2, and 0.2 % by DENV-3. Severe dengue affected 6.6 % of all cases, of which 32.3 % of the cases caused by DENV-2, 6.4 % of those caused by DENV-4, 4.5 % of those caused by DENV-1, and none of those caused by DENV-3. Severe dengue was found to be seven times more frequent among cases of DENV-2 than among those of the other serotypes.

**Conclusions:**

The present study found that cases of DENV-2 had a higher proportion of severe dengue than among those of DENV-1 and DENV-4. Consequently, early detection of serotypes circulating in the territory could be an important approach to prevent increasing numbers of severe outcomes during dengue outbreaks by predicting the health support needed for early diagnoses and treatment of dengue cases.

## Background

Dengue is a disease caused by a RNA virus of the family *Flaviviridae*, caused by four serotypes (DENV-1, DENV-2, DENV-3, and DENV-4) which are genetically and antigenically distinct. The virus is transmitted to humans by a bite from an infected mosquito of species *Aedes aegypti* and *Aedes albopictus* [1]. The clinical spectrum of dengue ranges from asymptomatic to severe presentations, with signs of increased vascular permeability, homeostasis disorders, and organ impairment. All serotypes are capable of inducing hemorrhage [2]. Determinants of dengue severity are not clear. Assumptions regarding pathogenesis focus on interactions between the dengue virus and its human host [3]. Virus strain, immune response to previous dengue infection, and genetic background of the host are factors that interfere in the propensity for worse outcomes [3]. Regarding dengue serotypes, their structural particularities affect the pathogenesis [4], since viral genetic components determine the virulence and ability to infect [5]. The magnitude of viral replication [4] also affects the severity of dengue [6–10]. Thereby, serotypes that tend to have higher replication rates induce a faster production of antibodies, causing more severe outcomes [7]. Therefore, the purpose of this study is to evaluate the influence of serotypes on clinical manifestations and outcomes of dengue.

## Methods

### Study design

A cross-sectional study was conducted using data obtained through the Information System for Notifiable Diseases (SINAN) on dengue cases that occurred in Vitória, Espírito Santo, Brazil, between 2009 and 2013. Vitória provides health care services with total public financing through the Unique Health System, which includes ambulatory care in the 28 health centers and in the two emergency units of the city, as well as laboratory services through the local public laboratory. Private health care plays a supplemental role in the health system. The municipality is a highly endemic area of dengue, with the four serotypes circulating in a period of five years and causing successive epidemics. More than 10 % of cases reported in the city presented specific laboratory tests for dengue confirmation, above the recommendation of the Brazilian Ministry of Health, in part due to the active surveillance by the Epidemiological Surveillance Service. In Brazil, suspected dengue cases must be reported to the Epidemiological Surveillance Service. In case of severe dengue, reporting has to be performed within 24 h. The data documented in the SINAN originate from standard report forms filled by physicians. The forms contain the description of clinical manifestations and the criteria of severe dengue. In addition, the Epidemiological Surveillance Service, which receives the report, controls the information which is documented on the report forms and the clinical condition of the patient during follow-up by contacting medical staff responsible for attending hospitalized cases, or the patient directly. Among the cases registered in SINAN (*n* = 30,027), all 485 (1.6 %) with information on serotyping were included in the analysis: 403 (83.1 %) with serotype determined by viral isolation and 82 (16.9 %) determined by polymerase chain reaction (PCR). Viral isolation was performed by inoculating cell cultures of *Aedes albopictus* (C6/36) and using indirect immunofluorescence method based on the reaction of the antibody specific for each of the four dengue serotypes with marking by fluorochrome, according to the technique established by Gubler et al. [11]. The PCR technique utilized was the reverse-transcriptase-polymerase chain reaction (RT-PCR), with amplification of cDNA obtained from dengue virus RNA, using specific initiators of DENV serotypes, following the technique described by Lanciotti et al. [12]. Both tests were performed in the period of viremia, before emergence of warning signs, minimizing the probability of selection bias. The patients submitted to these tests were selected systematically in sentinel sites from the public health sector, for surveillance purpose which did not influence the patient care. Among the cases included, data on gender, age, serotype, dengue outcome, and clinical manifestations were collected and evaluated.

### Definitions

The criteria of the Brazilian Ministry of Health were used to classify the cases as dengue fever (DF) and severe dengue (SD) [13]. Characterization of DF was determined by the presence of acute febrile illness lasting up to seven days, accompanied by at least two of the following signs or symptoms: headache, retro-orbital pain, myalgia, arthralgia, malaise, or rash [13]. The classification of SD combines the entities of dengue with complication (DWC) and dengue hemorrhagic fever (DHF). DWC cases demonstrated at least one of the following manifestations: neurological disorders (manifested by delirium, drowsiness, coma, depression, irritability, psychosis, dementia, amnesia, meningeal signs, paresis, paralysis, polyneuropathy, Reye syndrome, Guillain-Barré syndrome, and encephalitis), cardiac disorders (manifested by heart failure and myocarditis associated with myocardial depression, reduction in fraction ejection, and cardiogenic shock), hepatic failure (indicated by hepatomegaly, elevated level of hepatic enzymes and icterus), thrombocytopenia equal to or less than 20,000/mm^3^, gastrointestinal bleeding, cavity effusion (pleural effusion, pericardial effusion, ascites, checked by ultrasound or radiography), total leukocyte count equal to or less than 1,000/mm^3^ or death. DHF cases displayed all of the following characteristics: fever or recent history of fever for up to seven days, thrombocytopenia demonstrated by platelet count equal or less than 100,000/mm^3^, signs of hemorrhage (demonstrated by positive tourniquet test, hematuria, petechiae, menorrhagia, gingival bleeding, epistaxis, gastrointestinal bleeding), and signs of plasma leakage (hemoconcentration demonstrated by 20 % increase in the hematocrit over the baseline at admission or 20 % drop in hematocrit after appropriate treatment, cavity effusion, or hypoproteinemia) [13]. The age groups were classified as follows: children (0 to 9 years), adolescents (10 to 19 years), adults (20 to 59 years), and elderly (60 to 83 years).

### Statistical Analysis

The statistical analysis was carried out using R. Chi-square test, including Fisher exact test, was performed to compare the variables gender and serotypes between DF and SD, and clinical manifestations of SD between serotypes. Mann–Whitney *U* test was used to measure age differences between the outcome groups (DF and SD). The results were presented by *P*-values, and were defined as significant when below 0.05. Logistic regression was performed to adjust for gender, age group, and serotype to avoid confounding by these independent variables, considering men, elderly, and DENV-4 as reference categories. The choice of the reference category is based on the hypothesis and on the difference to be measured. In this case, the hypothesis is that DENV-2 increases the probability of severity. The nearest category in terms of frequency is DENV-4, what makes it the candidate for being the reference. The dependent variable was the clinical progress of dengue with dichotomous values: dengue fever and severe dengue.

### Ethical considerations

The Research Ethics Committee of the Health Sciences Center at Federal University of Espírito Santo (opinion number 881,909) and the Ethics Committee of the University of Munich (opinion number 231–15) approved the study protocol. Application of ethical consent form to patients was not considered necessary, since data were collected from the national Information System for Notifiable Diseases. The anonymity of patients and confidentiality of the secondary data is ensured by the researchers and institutions involved in the study. In addition, in this study no individual data is being presented. This approach has been supported by the above named ethics committees in Brazil and Germany.

## Results

The study population comprised 485 dengue cases, with a proportion of 46.4 % females and a median age of 26 years. Among them, 6.6 % presented SD. Among the cases with SD, the proportion of females was lower and the age was higher, although these differences were not significant (Table 1).

**Table 1:** Demographic characterization of study population

	Dengue fever	Severe dengue	Study population	*P*-value
Sample size (%)	453 (100)	32 (100)	485 (100)	N.A.
Proportion (%)	(93.4)	(6.6)	(100)	N.A.
Female (%)	215 (47.5)	10 (31.3)	225 (46.4)	0.08^a^
Proportion (%)	(95.6)	(4.4)	(100)	N.A.
Median of age (interquartile range)	26 (15–38)	30 (17–54)	26 (15–39)	0.15^b^

^a^Pearson chi-square test. ^b^Mann-Whitney *U* test

DENV-1 (77.3 %) was the predominant serotype, followed by DENV-4 (16.1 %) and DENV-2 (6.4 %). As only one dengue case was caused by DENV-3 (0.2 %), which occurred in a man who presented DF, DENV-3 could not be included in the analyses. Regarding SD, 4.5 % of cases with DENV-1, 32.3 % of cases with DENV-2, and 6.4 % of cases with DENV-4 presented this form of the disease. Cases caused by DENV-2 were more associated with SD (*P*-value < 0.01), whereas cases caused by DENV-1 presented a lower proportion of SD (*P*-value < 0.01) (Table 2).

**Table 2:** Proportion of cases with dengue fever and with severe dengue among patients diagnosed with different dengue serotypes

	Dengue fever	Severe dengue	Study population	*P*-value
Sample size (%)	453 (100)	32 (100)	485 (100)	N.A.
Proportion (%)	(93.4)	(6.6)	(100)	N.A.
DENV-1 (%)	358 (79.0)	17 (53.1)	375 (77.3)	<0.01^a^
Proportion (%)	(95.5)	(4.5)	(100)	N.A.
DENV-2 (%)	21 (4.6)	10 (31.3)	31 (6.4)	<0.01^a^
Proportion (%)	(67.7)	(32.3)	(100)	N.A.
DENV-3 (%)	1 (0.2)	0	1 (0.2)	1.00^b^
DENV-4 (%)	73 (16.1)	5 (15.6)	78 (16.1)	0.94^a^
Proportion (%)	(93.6)	(6.4)	(100)	N.A.

^a^Pearson chi-square test; ^b^Fisher’s exact chi-square test

In logistic regression, the proportion of SD among cases of DENV-2 was significantly higher than the SD among cases of DENV-4: adjusted OR = 7.42; 95 % CI 2.21–24.93. Cases caused by DENV-1 presented less SD than cases of DENV-4, although the difference was not significant (adjusted OR = 0.65; 95 % CI 0.23–1.88). Regarding demographic characteristics evaluated in logistic regression, the results were the following: the OR adjusted for females was 0.43 (95 % CI 0.19–1.01), considering males as a reference category, and taking elderlies as the reference category for age group, the OR adjusted for children was 0.19 (95 % CI 0.02–1.92), the OR adjusted for adolescents was 0.48 (95 % CI 0.12–1.87), and the OR adjusted for adults was 0.26 (95 % CI 0.07–0.96).

Positive tourniquet test and petechiae were the most common signs of hemorrhage in cases caused by DENV-1 and DENV-2. Hemoconcentration was the most common sign of plasma leakage in cases caused by DENV-2, and hypoproteinemia in cases caused by DENV-1. Cases caused by DENV-4 had hemoconcentration and cavity effusion as the main signs of plasma leakage, and hematuria and epistaxis as the main signs of hemorrhage. No clinical manifestation was significantly associated to any of the serotypes (Table 3).

**Table 3:** Clinical manifestations of severe dengue according to serotype

Clinical manifestation	DENV-1		DENV-2		DENV-4		All three serotypes
	n/N	*P*-value	n/N	*P*-value	n/N	*P*-value	n/N
Any neurological disorders (%)	1/17 (5.9)	1.00^b^	1/10 (10.0)	0.53^b^	0/5 (0)	1.00^b^	2/32 (6.3)
Any cardiac disorders (%)	1/17 (5.9)	1.00^b^	0/10 (0)	1.00^b^	0/5 (0)	1.00^b^	1/32 (3.1)
Any signs of plasma leakage (%)	6/14 (42.9)	0.90^a^	3/7 (42.9)	1.00^b^	2/4 (50.0)	1.00^b^	11/25 (44.0)
Hemoconcentration (%)	2/14 (14.3)	0.35^b^	3/7 (42.9)	0.30^b^	1/4 (25.0)	1.00^b^	6/25 (24.0)
Cavity effusion (%)	1/14 (7.1)	1.00^b^	0/7 (0)	1.00^b^	1/4 (25.0)	0.30^b^	2/25 (8.0)
Hypoproteinemia (%)	3/14 (21.4)	0.23^b^	0/7 (0)	0.53^b^	0/4 (0)	1.00^b^	3/25 (12.0)
Any sign of hemorrhage (%)	12/16 (75.0)	0.69^b^	6/8 (75.0)	1.00^b^	2/5 (40.0)	0.29^b^	20/29 (69.0)
Positive tourniquet test (%)	8/11 (72.7)	0.60^b^	3/4 (75.0)	1.00^b^	0/2 (0)	0.11^b^	11/17 (64.7)
Hematuria (%)	1/11 (9.1)	1.00^b^	0/4 (0)	1.00^b^	1/2 (50.0)	0.23^b^	2/17 (11.8)
Petechiae (%)	3/11 (27.3)	0.63^b^	3/5 (60.0)	0.27^b^	0/2 (0)	0.53^b^	6/18 (33.3)
Menorrhagia (%)	0/11 (0)	0.39^b^	1/5 (20.0)	0.28^b^	0/2 (0)	1.00^b^	1/18 (5.6)
Gingival bleeding (%)	2/12 (16.7)	1.00^b^	1/4 (25.0)	1.00^b^	0/2 (0)	1.00^b^	3/18 (16.7)
Epistaxis (%)	1/11 (9.1)	0.52^b^	1/4 (25.0)	1.00^b^	1/2 (50.0)	0.33^b^	3/17 (17.6)

^a^Pearson chi-square test; ^b^Fisher’s exact chi-square test

## Discussion

This cross-sectional study assessed the influence of different serotypes of dengue virus on the clinical outcomes of 485 laboratory cases of dengue infection detected in Vitória, Brazil, between 2009 and 2013. The results of the present study demonstrated that infections caused by DENV-2 were more associated with SD. In this study, gender and age did not influence the associations between the serotypes and the presentation of SD.

Previous studies confirm the present findings, showing an increased proportion of severe outcomes [14], such as DHF [2, 7, 15, 16], and dengue shock syndrome (DSS) [15, 17] in infections caused by DENV-2. Introduction of DENV-2 was a determinant factor for the emergence of SD in different global regions [3], and epidemics with a high number of severe hemorrhagic cases had DENV-2 as the predominant serotype [18, 19].

DENV-2 apparently played a crucial role in the emergence of severe cases in Vitória, considering the epidemiological overview seen in the period analyzed. In the municipality, the years with higher proportion of SD were 2009 (*n* = 5; 22.7 %), 2010 (*n* = 125; 23.4 %), and 2011 (*n* = 268; 10.9 %), when considering cases with laboratory confirmation. In the time series analyzed, 2009, 2010 and 2011 were the only years when DENV-2 was detected. In 2009, DENV-2 was the only serotype detected, but unfortunately, the number of laboratory tests performed was low in this period. Thereby, the restricted number of SD with laboratory confirmation impairs the link of this year with an increasing severity, especially that related to DENV-2 circulation. In 2010, DENV-2 was isolated from 52.9 % of samples with serotyping and was responsible for 88.9 % (*n* = 8) of severe cases with serotyping, providing evidence for its relevance in the emergence of SD during the epidemic.

The mechanism involved in DENV-2 virulence is not clear. One possible factor is the stimulatory effect of DENV-2 on nitric oxide production, causing toxic and inflammatory effects, inducing apoptosis in host cells [20]. Another factor responsible for the enhanced pathogenicity of DENV-2 is its efficient replication [7]. As a consequence, DENV-2 infections presented high viral load [14].

In the DENV-2 cases of the sample analyzed, milder hemorrhagic manifestations, such as petechiae and positive tourniquet test, were more common than serious ones, such as hematemesis, melena, hematuria, menorrhagia and epistaxis, as was found in a previous study [19]. It was not possible to generate evidence for severe manifestations being linked to DENV-2, such as plasma leakage [15, 21], cavity effusion [2], hypovolemic shock, internal hemorrhage [19], liver dysfunction [22], thrombocytopenia [21, 23] and hemoconcentration [21]. However, ten cases of SD caused by DENV-2 were present and larger sample sizes would be necessary to establish a statistically sound relation between DENV-2 and clinical manifestations.

DENV-3 was anteriorly related to severe forms of dengue [24], including DHF [16] and DSS [22, 25] and to other severe manifestations [25, 26], such as liver involvement [15, 22]. However, no conclusion can be drawn from the present study, as only one case was detected.

The present results suggest that those infected by DENV-1 evolved less frequently to severe outcomes. Less severe cases were linked to DENV-1 in previous investigations as well [22, 24], and plasma leakage was rarely observed [21]. In 2011, DENV-1 was isolated from 96.7 % (*n* = 355/367) of cases with serotyping in Vitória and was responsible for 88.2 % (*n* = 15/17) of the severe cases where serotyping was performed. Additionally, 2011 met other conditions related to increasing severity. In that period, there was a co-circulation of the serotypes DENV-1 and DENV-2, and probably a considerable number of secondary dengue infections occurred after years with wide circulation of DENV-2. Even with these two factors, DENV-1 presented better outcomes than other serotypes. Similarly to the present findings, Balmaseda et al. (2006) found DENV-1 to be associated with milder hemorrhagic manifestations, such as positive tourniquet test and petechiae [19]. The present study did not capture the presence of gastrointestinal symptoms in DENV-1, as reported by Thomas et al. [21].

There are discordant results in other studies. Fox et al. (2011) showed that DENV-1 and DENV-2 cases had similar chances to progress to DHF [27]. However, their study included only hospitalized cases, which may have introduced a bias. Yung et al. (2015) demonstrated that patients with DENV-1 had a higher chance of developing DHF than those with DENV-2 in Singapore. The DENV-2 Cosmopolitan genotype circulating in that period in Singapore [23] was different from the DENV-2 American/Asian genotype, circulating in Vitória [28], affecting the disease presentation. There is an association between the introduction of the Asian genotype in the Americas and the emergence of hemorrhagic cases. Up until 2003, all DENV-2 isolated from DHF cases on the American continent belonged to Asian genotype [29].

Cases with DENV-4 infection did not present increased or lower association with SD in the sample evaluated. Previous studies demonstrated that infections caused by DENV-4 presented less severe clinical manifestations [16, 21] and lower viral titers than other serotypes [30]. In Vitória, DENV-4 was detected for the first time in 2012. In 2013, DENV-4 was circulating in a highly susceptible population and caused the largest epidemic ever registered in Vitória, with more than 19,000 cases reported. Despite the impressive incidence, considering laboratory confirmed cases, SD affected only 7.6 % of cases in 2012 and 10.6 % of cases in 2013. Both years presented a lower occurrence of SD than 2009, 2010 and 2011, indicating the limited capacity of DENV-4 to cause SD. Halsey et al. (2012) showed the relation of DENV-4 and hemorrhagic cutaneous manifestations [31]. In the present study, no case with petechiae and positive tourniquet test was found in DENV-4 cases. However, the presence of only five severe cases caused by this serotype impaired the analysis on clinical manifestations.

The difference observed in serotypes association with severity is a concern in face of the recent approval of the first dengue vaccine in Brazil, since DENV-2 presented an efficacy of 42.3 % (varying from 14 % to 61.1 %) the lowest compared to other serotypes. [32]. A phase III trial for the vaccine has been conducted in Vitória since June 2011. However, no case of DENV-2 has been detected in the population since the beginning of the trial. Therefore, so far it is not possible to perceive the effect of the vaccination in this site in a scenario with DENV-2 circulation, regarding protection against dengue infection, emergence of severe cases and demand for hospitalizations. This issue will most likely be clarified in future, with the vaccine implementation and surveillance information.

The present study had some limitations. Potential influencing factors, such as secondary dengue infection, the sequence of serotypes responsible for the secondary infection [33], or co-morbidity, which could contribute to dengue severity, have not been assessed. As data collection was performed for surveillance purpose, it was dependent on professional precision by the involved health care workers when recording clinical information. Consequently, data on age and clinical manifestations were missing and could have been inaccurately documented. However, standardization of the report forms and the control of documented information by the Epidemiological Surveillance Service minimize information bias caused by misclassification. Determination of serotype was conducted in 485 systematically selected cases, who attended sentinel sites aiming at surveillance of circulating serotypes, which did not relate to patient care. Between 2009 and 2013, Vitória reported 30,027 suspected cases of dengue fever. In 1.6 % of them, serotyping was performed. Furthermore, the collection of blood occurred in the viremic phase, before the emergence of warning signs. As this was a test requested by the surveillance service during this period, it is unlikely that these cases were selected based on their clinical manifestations. Despite the fact that the study includes only 1.6 % of all dengue cases in the catchment area reported in the period, its sample was large enough to have sufficient power to detect the association between serotype and severe dengue. Dengue serotypes present genetic variations and some genotypes have a higher association with SD. Molecular studies of dengue in Vitória are necessary to define the genotypes circulating and their relation with severe epidemics.

Future investigations with prospective approaches in hyperendemic sites or multicenter settings could contribute to elucidate the role of different factors that influence the progression to severe dengue. Some of the factors that should be included are related to the virus, such as serotypes and genotypes, and others are related to the human hosts, such as demographic characteristics, co-morbidities and immunological status. Thereby, an integrated analysis of these factors could contribute to understand the complexity influencing severe dengue outcome.

## Conclusions

The present study found that cases of DENV-2 had a higher proportion of severe dengue than among those of DENV-1 and DENV-4. However, this finding was based on a small proportion (6.4 %) of DENV-2 cases among all analyzed samples and has to be confirmed in further studies. Surveillance of serotype circulation should occur permanently and intensively, since the early detection of serotypes circulating could be an effective tool to anticipate the number of severe outcomes during dengue outbreaks. With this information, health systems can be prepared to provide early detection of dengue cases, ensuring the follow up of patients and adequate treatment, preventing worse outcomes.

### Abbreviations

DF, dengue fever; DHF, dengue hemorrhagic fever; DSS, dengue shock syndrome; DWC, dengue with complications; SD, severe dengue
